# Impact of low-lipid environment on de novo lipid synthesis pathways in cancer cells

**DOI:** 10.1186/2051-1426-3-S2-P424

**Published:** 2015-11-04

**Authors:** Nousheen Zaidi

**Affiliations:** 1 University of the Punjab, Lahore, Pakistan

## 

Increased lipogenesis is a hallmark of a wide variety of cancers and is under intense investigation as potential anti-neoplastic target. Although brisk lipogenesis is observed in the presence of exogenous lipids, evidence is mounting that these lipids may adversely affect the efficacy of inhibitors of lipogenic pathways. Therefore, to fully exploit the therapeutic potential of lipid synthesis inhibitors, a better understanding of the interrelationship between *de novo* lipid synthesis and exogenous lipids and their respective role in cancer cell proliferation and therapeutic response to lipogenesis inhibitors is of critical importance. Here, we show that the proliferation of various cancer cell lines (PC3M, HepG2, HOP62 and T24) is attenuated when cultured in lipid-reduced conditions in a cell line-dependent manner, with PC3M being the least affected. Interestingly, all cell lines - lipogenic (PC3M, HepG2, HOP62) as well as non-lipogenic (T24) - raised their lipogenic activity in these conditions, albeit to a different degree. Cells that attained the highest lipogenic activity under these conditions were best able to cope with lipid reduction in term of proliferative capacity. Supplementation of the medium with very low density lipoproteins, free fatty acids and cholesterol reversed this activation, indicating that the mere lack of lipids is sufficient to activate *de novo* lipogenesis in cancer cells. Consequently, cancer cells grown in lipid-reduced conditions became more dependent on *de novo* lipid synthesis pathways and were more sensitive to inhibitors of lipogenic pathways, like Soraphen A and Simvastatin. Collectively, these data indicate that limitation of access to exogenous lipids, as may occur in intact tumors, activates *de novo* lipogenesis is cancer cells, helps them to thrive under these conditions and makes them more vulnerable to lipogenesis inhibitors. These observations have important implications for the design of new anti-neoplastic strategies targeting the cancer cell's lipid metabolism.

